# Intraspecific differences in metabolic rates shape carbon stable isotope trophic discrimination factors of muscle tissue in the common teleost Eurasian perch (*Perca fluviatilis*)

**DOI:** 10.1002/ece3.7809

**Published:** 2021-06-29

**Authors:** Kristin Scharnweber, Matilda L. Andersson, Fernando Chaguaceda, Peter Eklöv

**Affiliations:** ^1^ Department of Ecology and Genetics; Limnology Uppsala University Uppsala Sweden; ^2^ Department of Plant Ecology and Nature Conservation University of Potsdam Potsdam Germany; ^3^ Present address: Department of Aquatic Sciences and Assessment Swedish University of Agricultural Sciences Uppsala Sweden

**Keywords:** fractionation factors, metabolism, ontogeny, standard metabolic rate, tissue types, δ^13^C, δ^15^N

## Abstract

1. Stable isotopes represent a unique approach to provide insights into the ecology of organisms. δ^13^C and δ^15^N have specifically been used to obtain information on the trophic ecology and food‐web interactions. Trophic discrimination factors (TDF, Δ^13^C and Δ^15^N) describe the isotopic fractionation occurring from diet to consumer tissue, and these factors are critical for obtaining precise estimates within any application of δ^13^C and δ^15^N values. It is widely acknowledged that metabolism influences TDF, being responsible for different TDF between tissues of variable metabolic activity (e.g., liver vs. muscle tissue) or species body size (small vs. large). However, the connection between the variation of metabolism occurring within a single species during its ontogeny and TDF has rarely been considered.
2. Here, we conducted a 9‐month feeding experiment to report Δ^13^C and Δ^15^N of muscle and liver tissues for several weight classes of Eurasian perch (*Perca fluviatilis*), a widespread teleost often studied using stable isotopes, but without established TDF for feeding on a natural diet. In addition, we assessed the relationship between the standard metabolic rate (SMR) and TDF by measuring the oxygen consumption of the individuals.
3. Our results showed a significant negative relationship of SMR with Δ^13^C, and a significant positive relationship of SMR with Δ^15^N of muscle tissue, but not with TDF of liver tissue. SMR varies inversely with size, which translated into a significantly different TDF of muscle tissue between size classes.
4. In summary, our results emphasize the role of metabolism in shaping‐specific TDF (i.e., Δ^13^C and Δ^15^N of muscle tissue) and especially highlight the substantial differences between individuals of different ontogenetic stages within a species. Our findings thus have direct implications for the use of stable isotope data and the applications of stable isotopes in food‐web studies.

## Graphical abstract

In summary, our results emphasize the role of metabolism in shaping specific TDF (i.e., Δ^13^C of muscle tissue) and especially highlight the substantial differences between individuals of different ontogenetic stages within a species. Our findings thus have direct implications for the use of stable isotope data and the applications of stable isotopes in food‐web studies.

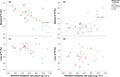

## 1 INTRODUCTION

Stable isotope analysis has become an established tool of ecologists for numerous applications, including research on ecosystem functioning (e.g., Mehner et al., 2016), animal migration (e.g., Hobson, 1999), ecophysiological processes (e.g., Gannes et al., 1998), and parasitism (e.g., Lafferty et al., 2008). Furthermore, it provides a useful tool for elucidating trophic interactions in food web research (Boecklen et al., 2011; Layman et al., 2012; Post, 2002). For these purposes, the ratio of carbon (^12^C/^13^C, expressed as δ^13^C values) and nitrogen (^14^N/^15^N, expressed as δ^15^N values) stable isotopes has been widely used. While δ^13^C values can be used to track the origin of the carbon source in organisms´ diet and the base of the food web, δ^15^N values are especially useful for determining the organisms´ trophic level (DeNiro & Epstein, 1978, 1981; Peterson & Fry, 1987). Combining these two approaches can provide information on resource and habitat use, thus allowing inference from the ecological niche of individuals, species, or communities (Bearhop et al., 2004; Martínez del Rio et al., 2009; Newsome et al., 2007).

For many applications of stable isotope analyses in ecology, estimates of the isotopic spacing between a consumer and its food (trophic discrimination) are needed. Trophic discrimination factors (TDF, Δ^13^C and Δ^15^N) represent the difference in values of δ^13^C (or δ^15^N) between the consumer and its diet. Most studies rely on average values for this parameter found in the literature, but the use of inaccurate TDF has been described as a major source of uncertainty in the use of mixing models to calculate the contributions of food items to the diet of a consumer (Phillips et al., 2014). Therefore, to allow precise interpretations of isotope data, appropriate TDF values obtained from relevant species‐specific trophic interactions are necessary (Martínez del Rio et al., 2009; Wolf et al., 2009).

TDF may vary considerably within and between species (Post, 2002), influenced, for example, by diet quality (Gaye‐Siessegger et al., 2003), feeding rates (Barnes et al., 2007), and the metabolic processes which shape the rate of diet incorporation (MacAvoy et al., 2005, 2006; Pecquerie et al., 2010). Metabolism describes the sum of all anabolic (synthesizing) and catabolic (degrading) processes of living organisms. In animals, metabolism is driven by the oxidation of organic molecules with the consumption of oxygen during cellular respiration. Part of this energy is used during anabolic processes to produce macromolecules (i.e., carbohydrates, proteins, or lipids). This will lead to an increase in tissue mass, resulting in growth, or to replacement of tissue, which are both important underlying processes shaping isotopic equilibration, potentially influencing TDF. Therefore, metabolic rate has strong implications for the rate at which isotopes are incorporated (Carleton & Martínez del Rio, 2010). It is generally acknowledged that more metabolically active tissues (e.g., liver) have faster turnover resulting in lower TDF due to the preferential incorporation of ^12^C in the excreted metabolites, compared with tissues with slower turnover (e.g., muscle) (Matley et al., 2016; McIntyre & Flecker, 2006; Xia et al., 2013). However, this framework has rarely been applied to the overall metabolism of an organism.

Fundamental differences in metabolic rates exist across the animal kingdom with higher mass‐specific metabolic rates in smaller species compared with larger ones (Kleiber, 1947). In addition to phylogenic differences in metabolism, metabolic rates also vary over ontogeny in individuals of the same species (Chabot et al., 2016; Wieser, 1984). To achieve high growth rates in younger individuals, these ontogenetic live stages are characterized by high metabolic rates (e.g., Hou et al., 2008; Yagi et al., 2010). In addition, a whole research field studies the consequences of metabolic differences between individuals irrespective of ontogenetic stages, and their influence on general behavior and performance (Biro & Stamps, 2010; Careau et al., 2008; Metcalfe et al., 1995), including social dominance, aggressive behavior, and activity levels (Reidy et al., 2000; Røskaft et al., 1986).

One component of an organism's metabolism is standard metabolic rate (SMR) which is the minimum metabolic rate needed for subsistence (Chabot et al., 2016; Hulbert & Else, 2004). Oxygen consumption, measured in a respirometer, is typically used as a proxy for SMR. This baseline is ecologically relevant in how it translates into differences in “maintenance costs” and thereby fitness, between conspecifics (Burton et al., 2011). Previous studies have been able to correlate individual metabolic rates with the differences of TDF found between species, sexes, and laboratory strains of endothermic animals with a high metabolism, such as birds and rodents (MacAvoy et al., 2006, 2012; Ogden et al., 2004), but this concept has not been broadened to ectothermic organisms with a slower mass‐specific metabolic rate, such as temperate fish.

In this study, we examined TDF for Eurasian perch (*Perca fluviatilis*), which is a ubiquitous fish in Europe and Asia (Froese & Pauly, 2020). It is the dominant predatory species in many aquatic habitats including freshwater (Mehner et al., 2007) and brackish systems (Ådjers et al., 2006), playing a fundamental role in structuring food webs (e.g., Bartels et al., 2016; Marklund et al., 2019; Svanbäck et al., 2015). Nonetheless, species‐specific stable isotope TDF for perch feeding on natural diets have not been established. Like many vertebrate predators, perch grow several orders of magnitude in body size over their lifetime (e.g., Hjelm et al., 2000), making this species an excellent model for studying the relationship between metabolism and TDF over ontogeny.

The motivation for this study was twofold. First, we wanted to experimentally derive Δ^13^C and Δ^15^N for different weight classes of Eurasian perch, to allow more accurate estimates of trophic positions, ecological niches, and other potential food‐web inferences from this common teleost. Second, we aimed at identifying the role of metabolic rate on TDF. We predicted that the higher mass‐specific standard metabolic rate of juvenile fish will result in lower TDF than those seen in adult perch.

## 2 MATERIAL AND METHODS

### 2.1 Fish collection, husbandry, and experimental design

Between 21 and 28 August 2018, we collected perch from lake Erken (59°50′09.6″N, 18°37′52.3″E) in Central Sweden by angling in littoral and pelagic habitats. We caught additional young‐of‐the‐year juveniles by beach seining. Using cooled and aerated boxes, we brought the fish to the aquarium facility at Uppsala University.

Fish were anesthetized using 60 mg/L benzocaine and weighted to the nearest 0.01 g. We grouped the fish according to their habitat of origin and the body weight at the beginning of the experiment into weight classes: <20 g (juveniles of approximately 4 g), 20–30 g, 30–40 g, and 40–50 g. Perch of the weight class 20–30 g were caught in both littoral and pelagic habitats. Due to potential differences, we analyzed this weight class habitat specifically. Three to nine perch individuals were placed together in 30 l aquaria (50 × 25 × 25 cm) with the bottom covered by a 3‐cm‐thick layer of sand. In order to maintain tanks with approximately equal‐sized shoals, tanks with fish from the same size class and same habitat were combined after periodic sampling. The room temperature of the facility was set at 15°C. Aquaria were equipped with a flow‐through system of fresh tap water. As the tap water became colder during the winter months (November–March), we noticed a drop in the tank water temperatures from 18 to 14℃. We fed the fish daily with commercially available chironomids, with an amount corresponding to approximately 15% of the individual wet weight per day. Unfortunately, we noticed strong variation in isotopic signatures of chironomids and were forced to switch suppliers to obtain more stable signatures in perch diet (final food: δ^13^C: −30.6‰ ± 0.5, δ^15^N: 14.0‰ ± 3.7; average ± *SD*, respectively). Using the new food, we started the feeding experiment to estimate TDF in perch on 10 October 2018 (day 0). We ended the trials between 12 (day 275) and 25 July 2019 (day 288), that is, the experiment ran slightly longer than 9 months. During this time, perch had approximately doubled their original body mass on average (132.7% weight gain ± 89.4, Table 1). This is an approximate value as we did not track the weight increase individually, but set initial weights as the average values of the respective weight class. We assumed perch to be in isotopic equilibrium with the chironomid diet based on the equations for the isotopic half‐life of vertebrate muscle tissue and liver reported in Vander Zanden et al. (2015) and the predictive turnover equations presented by Thomas and Crowther (2015). For both predictions, we assumed isotopic equilibrium in 4–5 times the half‐life (Thomas & Crowther, 2015). Before the fish were killed at the end of the experiment with an overdose of benzocaine, we conducted metabolic trials to obtain individual SMR (see the section below). We weighed the killed fish to the nearest 0.01 g and measured the total length to the nearest mm. We dissected a sample of the dorsal muscle tissue and the entire liver for stable isotope analyses and dried the tissue in a drying oven at 60℃ for 48 hr.

**TABLE 1:** Overview of averages (± standard deviations) of muscle and liver tissue TDF (Δ^13^C and Δ^15^N) for the different weight classes of perch, and standard metabolic rate (SMR), including sample sizes, weight at end of the experiment, and percent approximate change of weight (initial weight was not measured, but it was assumed to be the average of the respective weight classes)

Weight class at start (g)	Sample size	Habitat	Muscle Δ^13^C (‰)	Muscle Δ^15^N (‰)	Liver Δ^13^C (‰)	Liver Δ^15^N (‰)	SMR (mg O_2_ kg^−1^ hr^−1^)	Weight at end (g)	Percent approximate change of weight
<20 (juvenile)	4	Littoral	2.8 ± 0.2	2.6 ± 0.3	0.5 ± 0.5	1.2 ± 0.4	97.2 ± 8.9	16.6 ± 1.0	316.2 ± 24.2
20–30	4	Littoral	3.9 ± 0.3	1.1 ± 0.4	1.1 ± 0.2	0.8 ± 0.3	73.8 ± 6.3	56.2 ± 7.3	124.7 ± 29.1
20–30	11	Pelagic	3.6 ± 0.3	1.1 ± 0.3	1.4 ± 0.3	1.2 ± 0.5	83.7 ± 8.8	57.7 ± 11.0	137.3 ± 58.8
30–40	4	Pelagic	3.9 ± 0.4	1.1 ± 0.3	1.3 ± 0.3	1.1 ± 0.6	73.8 ± 3.8	40.4 ± 23.1	73.4 ± 45.3
40–50	5	Littoral	4.0 ± 0.3	1.1 ± 0.2	1.3 ± 0.1	0.8 ± 0.5	61.7 ± 8.3.	54.4 ± 21.2	56.0 ± 35.1

Over the course of the experiment, we killed a subset of five individuals every 6–10 weeks to observe the development of TDF over time. In total, we analyzed the Δ^13^C and Δ^15^N in muscle and liver for 48 and 47 individuals, respectively. In addition to the fish killed over the course of the experiment, 28 individuals were maintained for the entire 9‐month period and SMR measurements were taken for 26 of them.

The study was approved by the Uppsala Animal Ethic Committee with permit number: C59/15.

### 2.2 Stable isotope analyses and calculation of trophic discrimination factors

We ground ovendried tissue samples to a fine powder using a mortar and pestle, and transferred approximately 1 mg of the powder to tin capsules. Elemental and stable isotope analyses of carbon and nitrogen were conducted at the University of California, Davis Stable Isotope Facility, California, USA, using a PDZ Europa ANCA‐GSL elemental analyzer coupled with a PDZ Europa 20‐20 isotope ratio mass spectrometer (Sercon). We express our results using the δnotation, thus referring the ratios of samples to the international standards (Vienna Pee Dee Belemnite and air for carbon and nitrogen, respectively). Measurement error was 0.2‰ for ^13^C and 0.3‰ for ^15^N. As C/N in muscle tissue was low (3.2 ± 0.01; average ± *SD*), no lipid normalization was performed (Kiljunen et al., 2006). However, C/N in liver tissue (4.4 ± 0.6 average ± *SD*) exceeded the recommended ratio of 3.4 and we therefore used a mathematical lipid normalization on the δ^13^C values to account for the bias of carbon fractionation occurring during lipid synthesis (Kiljunen et al., 2006; Skinner et al., 2016; Sweeting et al., 2006). As recommended by Skinner et al. (2016), we used the percent lipid model introduced by Post et al. (2007), together with the normalization model described by Kiljunen et al. (2006).

We calculated TDF (i.e., Δ^13^C and Δ^15^N) for perch as follows:

$$\Delta X_{\text{perch}}=\delta X_{\text{perch}}‐\delta X_{\text{Chironomid}},$$

using average values for $\delta X_{\text{Chironomid}}$, where X stands for the heavy isotope of C (^13^C) and N (^15^N).

### 2.3 Measurements of standard metabolic rate (SMR)

Standard metabolic rate was measured using an intermittent flow respirometry system (Loligo Systems). A detailed description of the respirometry setup is described in Andersson et al. (2020). Briefly, our setup was comprised of four acrylic respirometry chambers, submerged in two aquaria (two chambers per tank) which contained water maintained at 18.1 ± 0.07℃ (average ± *SD*) and air‐stones to maintain oxygen at air‐saturation levels. Oxygen concentration in each chamber was measured using fiber‐optic optodes connected to flow‐through oxygen cells in conjunction with a Wiltrox4 oxygen meter (Loligo Systems). Chambers were size matched to each fish. Measurement loops for the trials consisted of a 180 s flush phase, a 30 s wait phase, and a 210 s measurement phase. Measurement loops for calculating background oxygen consumption rate (${\dot{M}O}_{2}$ (mgO_2_ hr^−1^)) consisted of a 180 s flush phase, a 30 s wait phase, and a 900 s measurement phase. The entire system was drained and flushed with bleach between trials to prevent the buildup of microbes over the course of the experiment. At the beginning of each trial, the system was refilled with aerated tap water that had been maintained at 18 ± 0.5℃ for a minimum of 24 hr.

Fish were fasted for approximately 24 hr before the start of the respirometry trial. Individuals were removed from their husbandry tank using a mesh net and transferred to the respirometer in a 9‐l bucket filled with aerated 18 ± 0.5℃ tap water. Fish were placed in the respirometer in the afternoon and remained in the respirometer for a minimum of 19 hr and 43 min. Following the trial, fish were killed with an overdose of benzocaine and frozen for stable isotope analysis. Measurements for background respiration were taken directly before and after each trial. AutoResp software (Loligo Systems, version 2.2.0), was used to calculate ${\dot{M}O}_{2}$ (mgO_2_/hr) from the linear decrease in dissolved oxygen over each measurement phase. Chamber‐specific background respiration was calculated by fitting a linear regression between all of the starting and ending background values with *R* ^2^ > 0.1 grouped by the chamber. The linear regressions between all values, as opposed to just values specific to each trial, were used so that measures with *R* ^2^ < 0.1 could be excluded, while still allowing for chamber‐specific background, which accounts for slight differences in calibration between each chamber. Each trial was adjusted for background respiration by subtracting fitted values estimating background respiration from measures of ${\dot{M}O}_{2}$ for each fish at each time point. Background corrected measures were divided by fish weight to calculate mass‐specific ${\dot{M}O}_{2}$ (mgO_2_ kg^−1^ hr^−1^), and any estimate of ${\dot{M}O}_{2}$ with an *R* ^2^ < 0.95 was removed prior to calculating SMR. SMR was calculated as the mean of the lowest 10% of ${\dot{M}O}_{2}$ measures (Andersson et al., 2020; Baktoft et al., 2016). R‐code for calculations of all metabolic measures is available on the data repository Zenodo (http://doi.org/10.5281/zenodo.4433723).

### 2.4 Statistical analyses

To analyze the variation in TDF between weight classes, we conducted four different ANCOVAs with subsequent Bonferroni post hoc comparisons using Δ^13^C and Δ^15^N, for the two tissue types, muscle and liver. We used final weight as a covariate with the attempt to adjust for the variation explained solely by growth (Fry & Arnold, 1982). Additionally, we conducted four linear regressions with the Δ^13^C and Δ^15^N, for muscle and liver tissues, respectively, as dependent variables and SMR as the predictor in order to analyze the relationship between TDF and SMR. We compared SMR between the different weight classes using an ANCOVA with subsequent Bonferroni post hoc comparisons, including final weight as a covariate. The assumptions of normal distribution and homogeneities of variances were met for all parametric analyses. We used IBM SPSS (version 25) for statistical analyses.

## 3 RESULTS

Over the course of the experiment, δ^13^C of muscle tissue gradually decreased from an average of −25.9‰ ± 0.3 (*SD*) measured after 8 weeks of feeding on a chironomid diet to −26.9‰ ± 0.4 (Figure S1a), while the δ^15^N of muscle tissue gradually increased from 14.2‰ ± 0.6 to 14.9‰ ± 0.2 (Figure S1b) during the same time interval. Compared with muscle tissue, lipid‐normalized stable isotope values in liver tissue were more depleted in ^13^C, leading to more negative values of δ^13^C (Figure S1c). δ^15^N of liver decreased from 16.5‰ ± 0.4 to 15.5‰ ± 0.3, and the liver tissue was generally more enriched in ^15^N compared with muscle tissue (Figure S1d).

**FIGURE 1:**
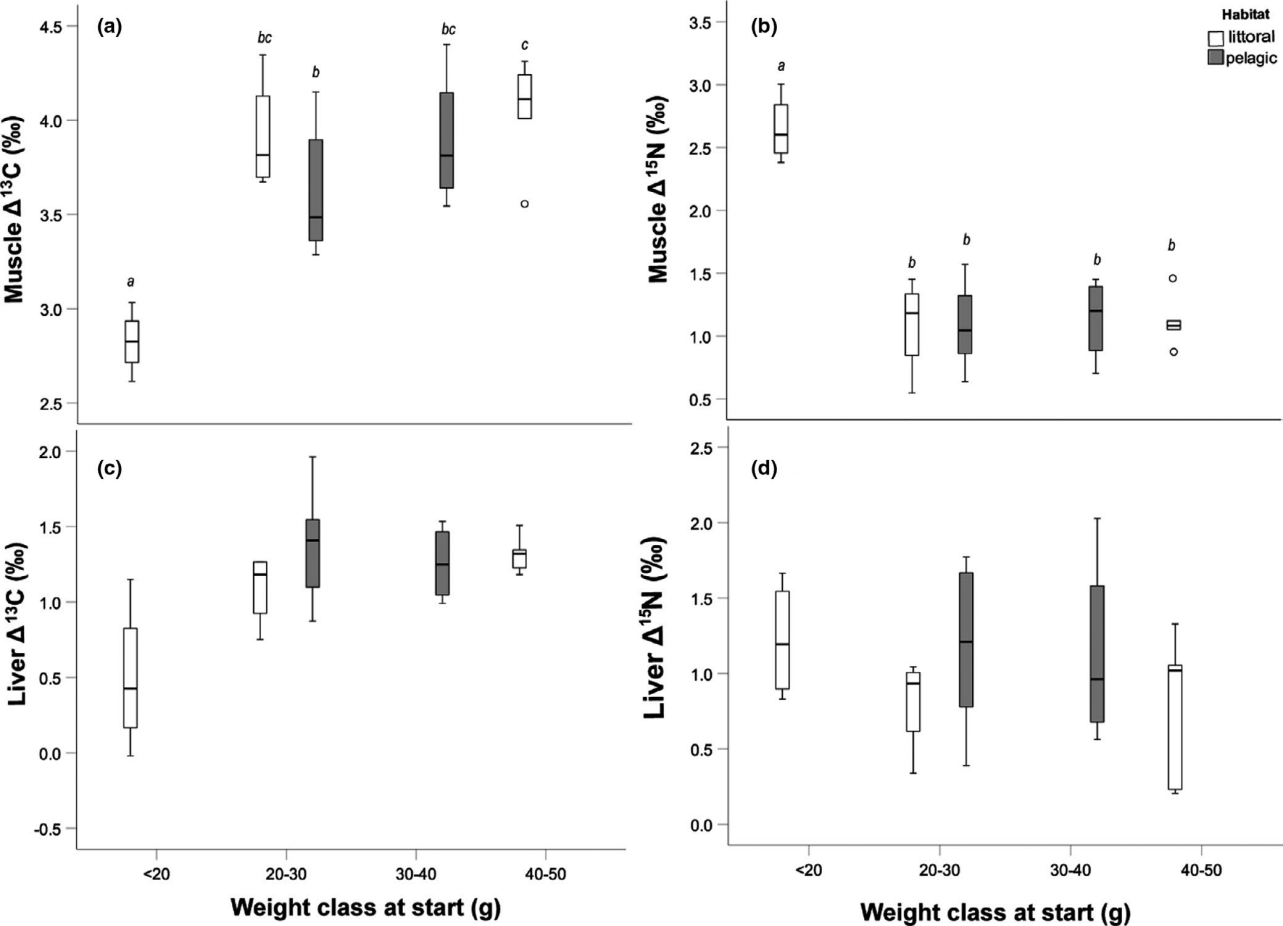
Δ^13^C and Δ^15^N of muscle and liver tissues of different weight classes in perch. Weight classes with the same letter are not significantly different (Bonferroni pairwise comparisons of ANCOVA with final weight as a covariate). Boxplots depict median, 25th and 75th percentile, and whiskers extend to maximum and minimum values, except for outliers (>1.5 times box height, represented by dots)

Δ^13^C of perch muscle ranged from 2.6‰ to 4.4‰ and for Δ^15^N from 0.5 to 3.0‰ (Table 1, Figure 1a,b). Δ^13^C of perch liver tissue ranged from 0.0‰ to 2.0 and for Δ^15^N from 0.2 to 2.0‰ (Table 1, Figure 1c,d). For muscle tissue, Δ^13^C differed significantly between weight classes (ANCOVA: *F*_5,28_:4.584, *p* = 0.008), with a significantly lower TDF in juvenile individuals (i.e., weight class <20 g at the start of the experiment) compared with all other weight classes. In addition, muscle Δ^13^C of 20–30 g individuals caught in the pelagic zone was significantly lower than that of 40–50 g individuals (Figure 1a). Δ^15^N in muscle tissue differed significantly between weight classes (ANCOVA: *F*_5,28_:18.365, *p* < 0.001), with significantly higher Δ^15^N observed in <20 g individuals compared with all other weight classes (Figure 1b). In this ANCOVA, final weight had a significant effect on the Δ^15^N of muscle tissue (ANCOVA: *F*_4,28_:7.403, *p* = 0.012), while this covariate was not significant for the Δ^13^C of muscle tissue. No significant differences were found in Δ^13^C and Δ^15^N of liver tissue between weight classes using final weight as a covariate (Figure 1c,d).

**FIGURE 2:**
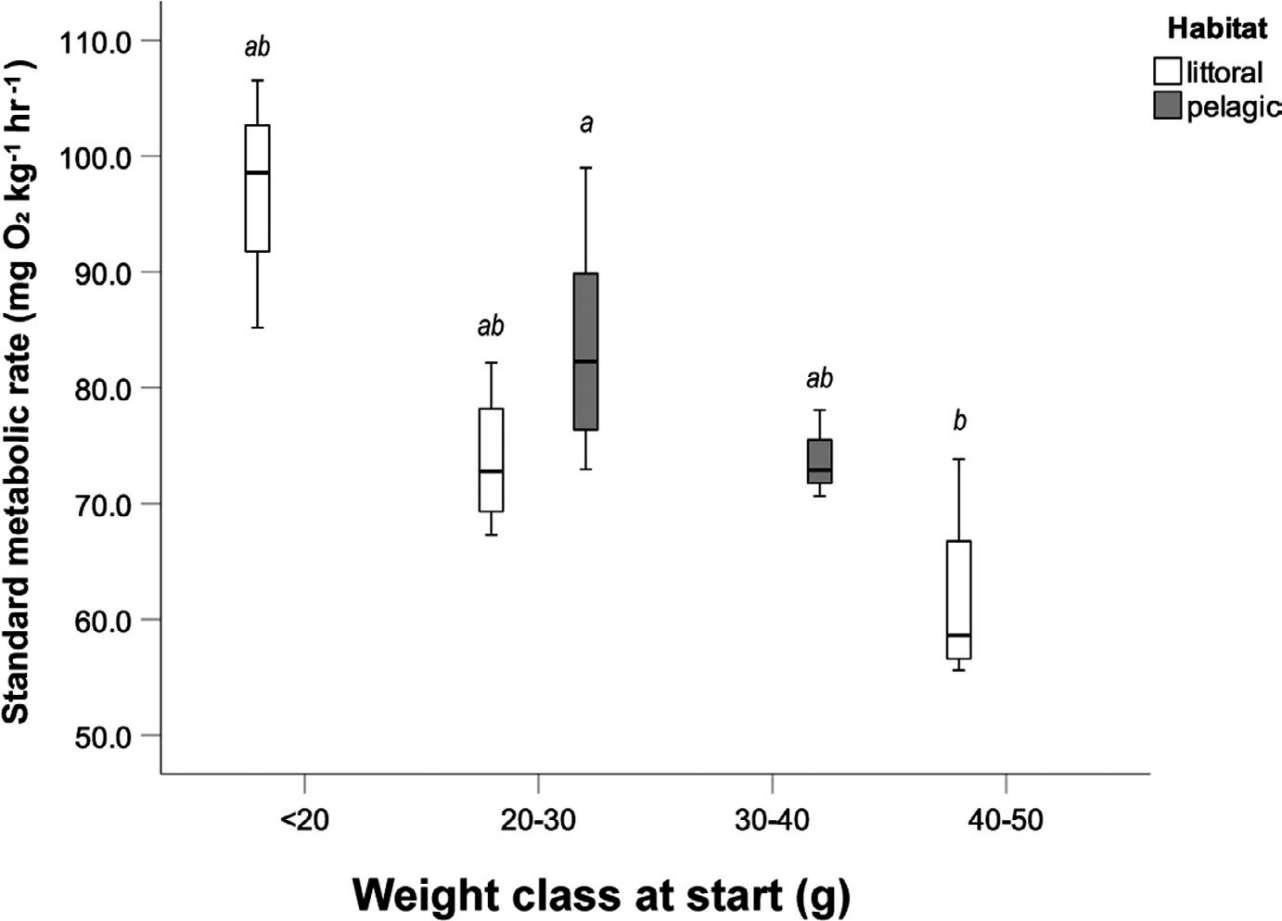
Standard metabolic rate (SMR) of different weight classes in perch. Weight classes with the same letter are not significantly different (Bonferroni pairwise comparisons of ANCOVA with final weight as a covariate). Boxplots depict median, 25th and 75th percentile, and whiskers extend to maximum and minimum values

SMR between the individuals varied substantially among perch groups and ranged from 55.6 to 106.5 mg O_2_ kg^−1^ hr^−1^ (Table 1). SMR differed significantly between perch of the different weight classes (ANCOVA: *F*_4,26_:4.685, *p* = 0.008) and final weight as a covariate was not significant. SMRs were highest in <20 g individuals, but Bonferroni‐adjusted pairwise comparisons showed that only differences between high SMR in 20–30 g individuals caught in the pelagic zone and the low SMR in individuals of 40–50 g were significant (Figure 2).

**FIGURE 3:**
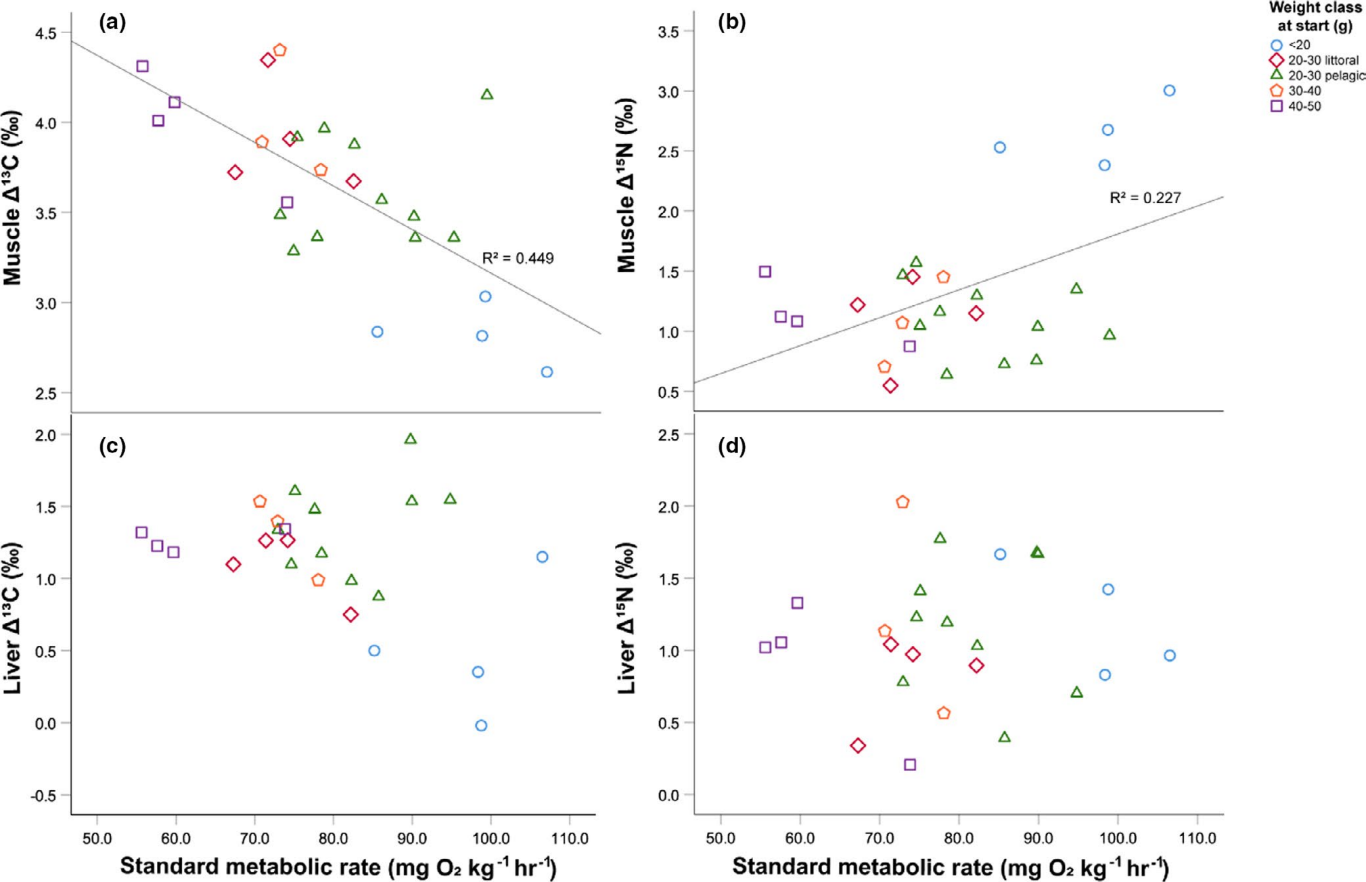
Relationship between standard metabolic rate (SMR) and Δ^13^C, and Δ^15^N of muscle and liver tissues in perch individuals, respectively. Colors depict different weight classes of perch at the beginning of the experiment. Regression lines (including regression coefficient *R* ^2^) in (a) and (b) indicate significant linear regressions between Δ^13^C and Δ^15^N and SMR in muscle tissue

For muscle tissue, linear regression showed that SMR had a significant negative effect on the Δ^13^C (*t* = −4.424, *p* < 0.001, Figure 3a) and a significant positive effect on Δ^15^N (*t* = 2.657, *p* = 0.014, Figure 3b), respectively. In contrast, this relationship was not significant for Δ^13^C or Δ^15^N in liver tissue (Figure 3c,d).

## 4 DISCUSSION

Metabolic rate can be viewed as the most fundamental biological rate, explaining the pace at which organisms take up, transform, and expend energy (Brown et al., 2004). Thus, we also predict that the individual metabolic rate would profoundly shape TDF. To the best of our knowledge, this framework has only been applied to the study of TDF in small mammals (i.e., mice and rats: MacAvoy et al., 2006, 2012), or birds (Ogden et al., 2004). However, in temperate ectothermic teleosts, where mass‐specific metabolic rate is comparably slower, this process was assumed to be negligible. This view needs a revision, as our results show a clear and gradual relationship between TDF of muscle tissue (i.e., Δ^13^C and Δ^15^N) and metabolic rate on the individual level. Thus, our results highlight that individual metabolic rate could be one of the factors explaining variable TDF values within a single species. However, we could not detect any association between metabolic rate and TDF in liver tissue. Differences in metabolic rates and therefore TDF were especially pronounced between small and large perch. Thus, in accordance with Herzka (2005), our results highlight the need for establishing different TDF for specific ontogenetic stages to allow more precise interpretation of isotopic data.

Trueman et al. (2005) reported differences in individual growth rates of Atlantic salmon (*Salmo* *salar*) to be associated with variable TDF. The authors suggested that this pattern could be explained by intraspecific differences in metabolism, but direct measurements of metabolic rates were not included. In many studies of TDF in ecothermic species, isotopic change has been attributed to growth rather than metabolism (Bosley et al., 2002; Hesslein et al., 1993), but see (Herzka et al., 2001; Sun et al., 2012; Tarboush et al., 2006). Unfortunately, we are not able to separate the isotopic change into contributions of growth and metabolic rate (sensu Fry & Arnold, 1982) as we did not track the precise individual weight increase. We approached this issue by including the final weight as a covariate in our models. Here, the strongest differences in TDF could be detected comparing juvenile with adult perch. Along with the highest SMR, juveniles also had the strongest approximate weight increase (Table 1). When measuring individual metabolic rate immediately before assessing their TDF, we found fine‐scale differences that correlated strongly with Δ^13^C and Δ^15^N of muscles indicating that changes in metabolic rates could translate into variations in TDF (Boecklen et al., 2011; Kleiber, 1932, 1947).

Results of post hoc comparisons of SMR of perch with an initial weight of 20–30 g caught in different habitats (pelagic and littoral) were not significant, but when analyzed separately, a significant difference appears with pelagic having a higher SMR (*t* test: *t*_7.633_ = −2.406; *p* = 0.044), similar to the previous results (M. L. Andersson et al., unpublished data). Generally, it is known that such intraspecific differences in metabolic rates within adult individuals exist in many species, including fish (Biro & Stamps, 2010). However, less is known about differences between individuals living in different habitats. In many Swedish lakes including Erken, which is the origin of the perch used in this study, littoral and pelagic perch of the intermediate class size differ in their individual specialization for respective food items, which is even translated into adaptations of their morphology. While pelagic perch predominately ingest pelagic zooplankton and have a more streamlined body form, littoral perch include benthic macroinvertebrates in their diet to a higher degree and are characterized by a deeper body (Marklund et al., 2019; Svanbäck & Persson, 2004). Potentially, habitat‐specific differences in activity levels could be related to the differences found in SMR (Myles‐Gonzalez et al., 2015; Watz et al., 2015). Pelagic perch need to be endurance swimmers in order to catch the smaller fast‐moving prey, while littoral perch forage on larger prey items with lower mobility (Svanbäck & Eklöv, 2004). Further research is needed to resolve the underlying causes for the differences found in SMR between littoral and pelagic perch. Interestingly, average Δ^13^C in muscle tissue of 20–30 g pelagic perch was lower than that of littoral perch of the same weight class (Table 1), but this difference was not significant. Thus, our data suggest a trend that the inverse relationship (i.e., elevated SMR leads to lower Δ^13^C) holds true not only between juveniles and adults, but also between the habitat‐specific individuals of the same weight class.

While the effect of SMR was strong for Δ^13^C and Δ^15^N in muscle tissue, we did not observe any relationship between SMR and TDF of liver tissue, indicating that individual metabolic rate has a stronger effect on tissue types with slower isotopic turnover. Generally, liver had lower TDF Δ^13^C: 1.1‰ ± 0.4; Δ^15^N: 1.1‰ ± 0.5) than TDF of muscle tissues Δ^13^C: 3.7‰ ± 0.5; Δ^15^N: 1.3‰ ± 0.6), showing relatively little change in the isotope values between consumer and prey. Our results are in line with the findings of other studies on tissue‐specific differences in TDF in fish (Buchheister & Latour, 2010; Matley et al., 2016), and the observed pattern might be due to different biochemical composition of the tissue types, for example, the abundances of specific amino acids (Pinnegar & Polunin, 1999).

Our values of Δ^15^N in muscle tissue are relatively low compared to the highly cited average value of 3.4‰ (Post, 2002). However, variation in Δ^15^N between taxa is large, which is primarily attributed to different nitrogen assimilation and excretion modes (Gaye‐Siessegger et al., 2003; Vanderklift & Ponsard, 2003). Thus, fractionation is typically low when the C:N ratio of the diet is also low, as it was for our chironomid diet (4.7 ± 0.2) (Vanderklift & Ponsard, 2003). For example, McCutchan et al. (2003) reported Δ^15^N of 1.4 ± 0.21 in consumers raised on an invertebrate diet, whereas consumers raised on a high‐protein diet showed a Δ^15^N of 3.3 ± 0.26. Protein is the principal source of energy for perch, which are ammoniotelic organisms, and thus characterized by a relatively high N use efficiency that is linked to lower Δ^15^N (Trueman et al., 2005). Furthermore, a previous study of perch stoichiometry reported that C:N ratio of perch varied with size, indicating that stoichiometric demands vary over ontogeny (Vrede et al., 2011). This could potentially affect assimilation and excretion rates and could have contributed to the variation of TDF between size classes as we have observed in this experiment, but further experiments are needed to assess this factor.

In contrast, our derived values of Δ^13^C for muscle tissue were rather high compared to the typically assumed value of 0.3‰ (Post, 2002) or 0.4‰ (McCutchan et al., 2003), though previous studies have reported similarly high values (e.g., Barnes et al., 2007; Busst & Britton, 2016; Pinnegar & Polunin, 1999). Vollaire et al. (2007) reported Δ^13^C of 4.02 ± 0.13‰ for juvenile perch feeding on artificial diet. A potential reason for variation in Δ^13^C could be a process termed “isotopic routing”, where resource constituents, such as proteins, lipids, and carbohydrates are allocated to different tissue types (Schwarcz, 1991). Altogether, we thus agree with Wolf et al. (2009) and acknowledge that further studies are urgently needed to understand variation of Δ^13^C in fish.

Variation in Δ^15^N, specifically in liver tissue, was higher than that in Δ^13^C. We assume that this variability can be attributed to the fact that the δ^15^N of the diet was rather high (14.0‰ ± 3.7). Commercially raised chironomids are maintained in large flumes and cannibalism might occur which would result in higher δ^15^N of individual organisms. Another aspect that could potentially influence the variation in Δ^15^N, but also in Δ^13^C is the individual food intake, which was shown to influence TDF (Barnes et al., 2007; Bosley et al., 2002). In our study, perch shoals were fed at high rations (approximately 15% of the individual wet weight per day), and leftover food was removed. However, this does not imply that all fish individuals fed until satiation. Strong hierarchies exist in perch shoals (Magnhagen, 2012) and were observed in some of our tanks. Dominance behavior of single individuals might have prevented subordinates the access to food. This artefact of the experimental design adds to the previously mentioned confounding aspects and highlights again the complexity and difficulty involved in experimentally assessing general and widely applicable TDF.

## 5 CONCLUSION

In conclusion, our data emphasize the role of metabolic rate in shaping specific TDF (i.e., Δ^13^C and Δ^15^N of muscle tissue). Particularly, our results highlight the substantial differences between individuals of different ontogenetic stages within a species.

## CONFLICT OF INTEREST

The authors declare that they do not have any conflict of interest.

## AUTHOR CONTRIBUTIONS

**Kristin Scharnweber:** Conceptualization (lead); Formal analysis (lead); Investigation (lead); Methodology (lead); Project administration (lead); Writing‐original draft (lead); Writing‐review & editing (lead). **Matilda L**. **Andersson:** Conceptualization (lead); Formal analysis (lead); Funding acquisition (equal); Investigation (lead); Methodology (lead); Project administration (lead); Writing‐review & editing (equal). **Fernando** **Chaguaceda:** Funding acquisition (equal); Investigation (equal); Methodology (equal); Writing‐review & editing (equal). **Peter** **Eklöv:** Formal analysis (equal); Funding acquisition (equal); Investigation (equal); Methodology (equal); Resources (lead); Writing‐review & editing (equal).

## ETHICAL APPROVAL

All applicable institutional and national guidelines for the care and use of animals were followed according to ethical permission C59/15, Uppsala Djurförsöketiska Nämnd.

### OPEN RESEARCH BADGES

This article has been awarded Open Data and Open Materials Badges. All materials and data are publicly accessible via the Open Science Framework at http://doi.org/10.5281/zenodo.4433723.

## DATA AVAILABILITY STATEMENT

Data and R scripts are available from the Zenodo Digital Repository (http://doi.org/10.5281/zenodo.4433723).

## Supporting information

Figure S1
