# Supplementary material for: Intraspecific differences in metabolic rates shape carbon stable isotope trophic discrimination factors of muscle tissue in the common teleost Eurasian perch (Perca fluviatilis)
Source: Ecol Evol. 2021 Jun 29;11(14):9804–14. doi: 10.1002/ece3.7809 (PMC8293782; doi:10.1002/ece3.7809)
Supplement: Supplementary file 1 — Figure S1 [file ECE3-11-9804-s001.docx]

**Supplementary Material**

**Figure S1:** Values of δ^13^C and δ^15^N in muscle and liver tissue respectively of pelagic perch of weight class 20-30 g sacrificed over the course of the experiment. Values of liver δ^13^C were lipid-normalized.

**
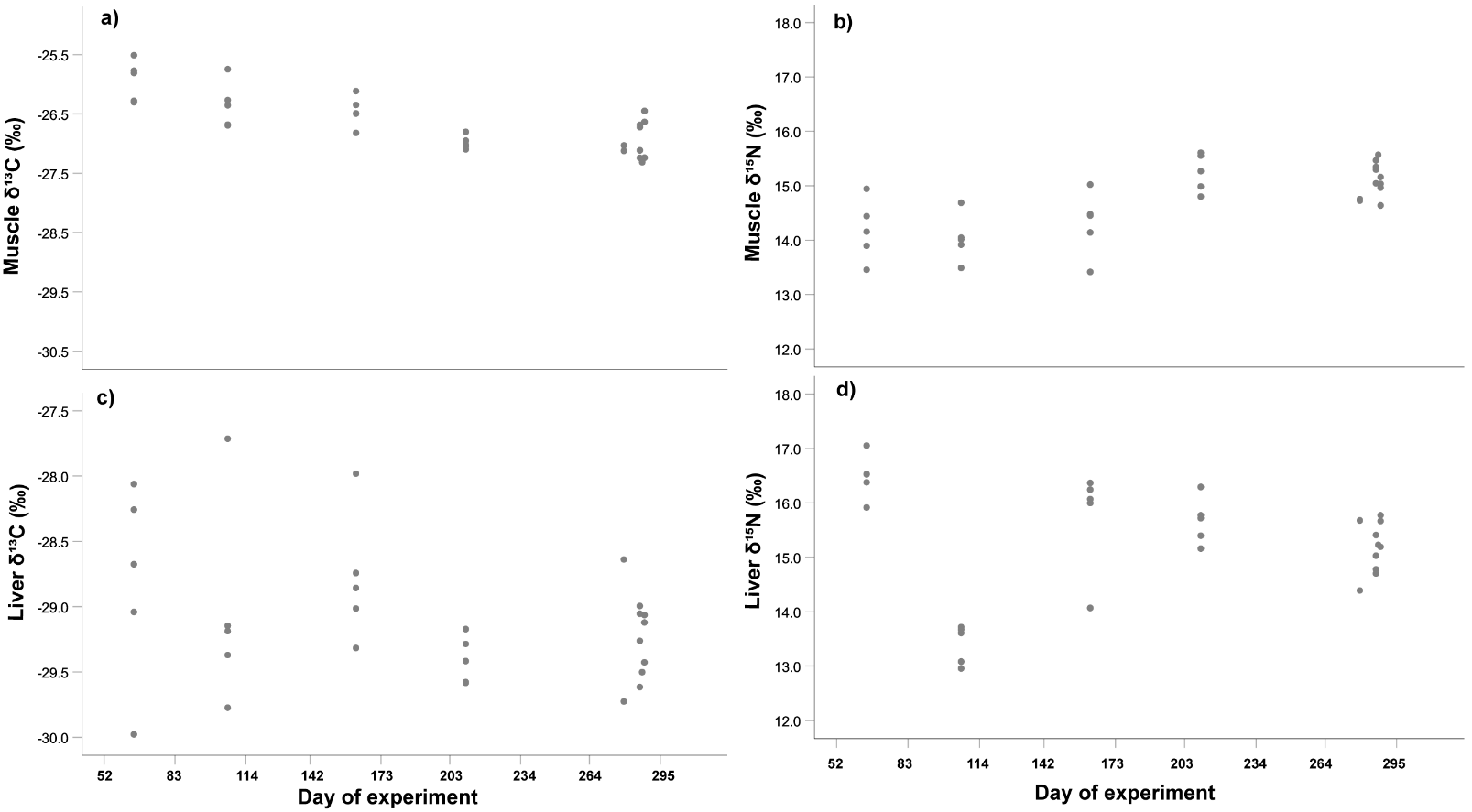
**
